# Effects of peppermint essential oil and artifier on growth performance, carcass characteristics and nutrient digestibilities in broiler chickens fed with low energy diets

**DOI:** 10.1016/j.vas.2024.100354

**Published:** 2024-04-21

**Authors:** Shokoufe Ghazanfari, Ayub Shiri Ghzghapan, Shirin Honarbakhsh

**Affiliations:** Department of Livestock and Poultry Sciences, Faculty of Agricultural Technology (Aburaihan), University of Tehran, Pakdasht, Tehran, Iran

**Keywords:** Peppermint essential oil, Emulsifier, Broiler chickens, Feed conversion ratio, Nutrient utilization

## Abstract

The aim of this experiment was to investigate the effects of peppermint essential oil (PEO) and an emulsifier called artifier on growth performance, carcass characteristics, and nutrient digestibility of broiler chickens fed low-energy diets. A total of 240 one-day-old male Ross 308 broiler chickens were divided into five treatments: control, low-energy diet, low-energy diet + 150 ppm PEO, low-energy diet + 300 ppm artifier, and low-energy diet + 150 ppm PEO + 300 ppm artifier. Each treatment was replicated four times in a completely randomized design. The chickens were fed these treatments during the starter, grower, and finisher periods for a total of 42 days. The results indicated that broiler chickens receiving the low-energy diet supplemented with PEO + artifier had similar body weight gain, feed conversion ratio, and breast percentage, but exhibited significantly lower abdominal fat percentage compared to those fed the control diet (*P* < 0.05). Furthermore, birds receiving PEO and artifier in low-energy diets, either individually or in combination, demonstrated higher digestibility of dry matter and fat during the starter and grower periods in comparison to those receiving the low-energy diet without any additives (*P* < 0.05). Over the entire rearing period, the simultaneous inclusion of 150 ppm PEO and 300 ppm artifier in the low-energy diet resulted in comparable growth performance to the control diet. Additionally, the concurrent use of PEO and artifier in the low-energy diet for broiler chickens led to a decrease in abdominal fat, increased digestibility of fat and dry matter, and enhanced nutrient utilization compared to the control diet.

## 1 Introduction

Advancements in genetic improvements in the poultry industry have resulted in the breeding of fast-growing chicks that require high-energy diets for optimal growth. However, these high-energy diets can lead to problems such as fatty liver in chicks. Increased dietary energy intake, combined with constant protein levels, can cause excessive fat deposition in the chicks' bodies (Buyse et al., 2001). This undesirable fat conversion reduces feed efficiency and economic performance for poultry producers. Additionally, increased carcass fat is undesirable for slaughterhouses as it is a non-consumable product (Nobakht & Aghdam Shahriar, 2010). It is worth noting that broiler chickens have a slightly slow optimal secretion mechanism of fat-digesting enzymes, which occurs around 10–16 days. Therefore, broilers that grow rapidly may struggle to cope with the high-fat content of their feed, leading to potential issues (Zhang et al., 2011).

To address the issues associated with high-energy diets, reducing the energy content of the diet is a suitable solution. While reducing the energy content of the diet may seem like a suitable solution, it can have adverse effects on feed conversion ratio, growth performance, and nutrient digestibility. To counteract these effects, additives are used, with antibiotics like virginiamycin being a common choice. Antibiotics improve digestion and absorption, enhance energy utilization, and promote poultry performance. However, antibiotic use has drawbacks, such as the emergence of drug-resistant bacteria and the presence of residual drugs in poultry products, leading to legal restrictions (Chen et al., 2019). In the pursuit of affordable and healthy food production, alternatives to growth-promoting antibiotics have been extensively researched. These alternatives encompass a wide range of options, including organic acids, herbal extracts, enzymes, antimicrobial peptides, and probiotics, among others. The objective of these alternatives is to provide similar benefits in terms of growth promotion and feed efficiency without the associated drawbacks of antibiotics (Tamizi Jooneghani et al., 2016). By implementing these alternatives, it is possible to address the reduction in antibiotic use in poultry production, ensuring the production of affordable and healthy food while minimizing any negative effects on animal and public health.

Peppermint essential oil, due to its active compounds such as phenols, polyphenols, terpenoids, alkaloids, lectins, and polypeptides, possesses antimicrobial and immune-stimulating effects, gastrointestinal stimulant properties, reduction of blood fat and cholesterol concentrations, antioxidant and anthelmintic properties, and ultimately acts as a growth promoter (Rezvani et al., 2019). Research results indicate that using a 0.3 % peppermint extract in the drinking water of broiler chickens increases performance and carcass yield while reducing abdominal fat deposition (Nanekarani et al., 2012). The investigation of the effects of peppermint essential oil on the performance and digestibility of broiler chicks showed that the use of 200 ppm peppermint essential oil increased protein digestibility compared to other treatments (Khodambashi Emami et al., 2012). Regarding the role of peppermint essential oil, it appears that it can potentially serve as an alternative to antibiotic growth promoters by acting as an antibacterial agent in the digestive tract. The antimicrobial properties of peppermint essential oil suggest that it may help control bacterial growth in the gastrointestinal system of broiler chickens, leading to improved performance and nutrient digestibility and reduced abdominal fat deposition (Nanekarani et al., 2012). Furthermore, feeding rats peppermint for 8 weeks (Platel & Srinivasan, 2000; 10 g/kg) resulted in an increased rate of bile acid secretion. Additionally, a study by Rao et al. (2003) reported that direct contact with peppermint enhanced the activity of pancreatic lipase and amylase in rats. In conclusion, peppermint essential oil improves fat digestion.

Another additive that enhances fat digestion in birds are emulsifiers. The most significant impact of using emulsifiers in the diet is aiding in the digestion and absorption of fats. Improved metabolizable energy and crude protein utilization efficiency when using an emulsifier supplement in the diet indicate the positive effect of emulsifiers on fat and other nutrient digestion and absorption (Sugawara et al., 2001). During the starter growth period, due to insufficient production of lipase enzymes, incomplete reabsorption of biliary salts, and inadequate production of fat-carrying proteins, fat digestion capacity decreases. Therefore, to improve fat digestion in bird diets, additives such as emulsifiers can be used (Zhang et al., 2011). Emulsifiers contribute to increased formation of emulsion droplets, stimulation of micelle formation, increased concentration of monoglycerides in the intestine, facilitation of nutrient transfer across membranes, and consequently, improved absorption of nutrients and energy utilization. Artifier is considered a synthetic emulsifier that contains various compounds of phospholipids and an emulsifier called polyethylene glycol ricinoleate (PEGR). Therefore, it appears that it can potentially assist in the more efficient digestion and absorption of dietary fats (Sugawara et al., 2001).

Researchers have shown that simultaneous use of peppermint essential oil and emulsifiers in pig diets reduces the dietary energy level and increases nutrient absorption without significantly affecting the feed conversion ratio (Li et al., 2017). Considering the various metabolic activities of peppermint essential oil and emulsifiers (artifier) in low-energy diets for broiler chicks is suggested to have potential benefits in terms of improving performance characteristics, reducing the negative effects of low-energy diets, and enhancing nutrient digestibility. A comparison with the control diet (with energy level matching the requirements of Ross 308 strain) is suggested to evaluate the impact of peppermint essential oil and emulsifiers on growth performance, carcass traits, nutrient digestibility in the early stages, and overall growth of broiler chicks.

## 2 Materials and methods

The Animal Ethics Committee of the Animal Science Research Institute of Iran and the Animal Care Committee of the Department of Animal Science, University of Tehran, approved all experimental procedures conducted in this study. Peppermint essential oil, sourced from Barij Essence Company (Kashan, Iran) was prepared using the following active ingredients: menthol (39.81 %), menthone (19.55 %), neomenthol (8.83 %), methyl acetate (8.64 %), and 8-cineole (5.81 %). Artifier, obtained from Golbar Navid Bahar Company (Tehran, Iran). Artifier is considered a polar emulsifier that contains four types of lysophospholipids (lysophosphatidylcholine, lysophosphatidic acid, lysophosphatidylethanolamine, lysophosphatidylinositol) as well as an emulsifier called polyethylene glycol ricinoleate. Peppermint essential oil and artifier were included in the diets as feed additives. The essential oil was in liquid form, and the required amount (150 ppm) was mixed with soybean oil before being added to the diets. Artifier, on the other hand, was initially mixed with one kilogram of ground corn and then added to the diets.

### 2.1 Experimental treatments, broiler chicken and diets

A total of 240 one-day-old male broiler chicks of Ross 308 strain with an average weight of 42 g were used in a completely randomized design for 42 day of age. The experiment included 5 treatments, 4 replications, and 12 chicks per replicate. The experimental treatments consisted of: 1. Control treatment (with energy level matching the requirements of Ross 308 strain), 2. Low-energy diet treatment (150 kcal per kg less than the requirements of Ross 308 strain), 3. Low-energy diet + 150 ppm peppermint essential oil, 4. Low-energy diet + 300 ppm artifier, and 5. Low-energy diet + 150 ppm peppermint essential oil + 300 ppm artifier. The broiler chickens were vaccinated for infectious bronchitis (Nobilis IB 4/91; Merck Animal Health, Boxmeer, the Netherlands) on the first day, avian influenza (Nobilis Infuenza TRT; Merck Animal Health) on day 7 and Newcastle disease (Nobilis G + ND; Merck Animal Health) on days 18 and 28. The feeding regimen for the broiler chickens consisted of three different diets: starter (day 0 to 10), grower (day 11 to 24), and finisher (day 25 to 42). The broiler chickens had *Ad libitum* access to both feed and water throughout the study. The temperature and humidity levels were carefully regulated and maintained within the optimal range for the birds' well-being. During the initial three days, the broiler chickens were exposed to a constant 24-h light period. From day 4 onwards, the photoperiod was adjusted to 23 h of light and 1 h of darkness per day until day 42. Throughout the study, the ventilation rate in the chicken house remained consistent. Additionally, the initial house temperature of 32 °C was gradually reduced to reach 20 °C by day 42. Freshly prepared diets in mash form were provided on a weekly basis. The experimental diets were formulated using corn-soybean meal based on different periods using UFFDA software (UFFDA Software Guide, 2008), and to meet the recommended nutrient requirements of commercial Ross 308 strain (except for energy) for three different periods: starter (0 to 10 days of age), grower (11 to 24 days of age), and finisher (25 to 42 days of age) (Table 1) (Aviagen, 2018). Chicks were transferred to a room with 20 floor pens (each with a length and width of 150 cm).

**Table 1:** Basal and low energy diets at different periods including starter (0 to 10 days), grower (11 to 24 days) and finisher (25 to 42 days) periods.

Ingredients (%)	Starter		Grower		Finisher	
	Low energy diet	Control diet	Low energy diet	Control diet	Low energy diet	Control diet
Corn	51.73	56.00	58.93	63.10	64.23	67.50
Corn gluten	0.7	3.5	0.6	2.8	0	2.5
Soybean meal	40.2	35.5	32.0	28.3	28.0	24.5
Concentrate A or B^a^	2.5	2.0	2.5	2.5	2.5	2.0
Soybean oil	0.3	0.4	0.5	0.7	0.5	0.9
Sand	0	0	1.3	0	1.0	0
Limestone	1.8	0.68	1.4	0.68	1.3	0.68
DiCalcium phosphate	1.6	0.8	1.6	0.8	1.3	0.8
Salt	0.35	0.30	0.35	0.30	0.35	0.30
L-lysine	0.07	0.07	0.07	0.07	0.07	0.07
DL- methionine	0.25	0.25	0.25	0.25	0.25	0.25
Vitamin premix^b^	0.25	0.25	0.25	0.25	0.25	0.25
Mineral premix^b^	0.25	0.25	0.25	0.25	0.25	0.25
Nutrient composition						
Metabolizable energy (kcal/kg)	2850	3000	2900	3050	2950	3100
Crude protein (%)	23.0	23.0	20.0	20.0	18.3	18.3
Fat (%)	2.30	2.50	2.30	2.60	2.64	2.79
Calcium (%)	1.06	0.90	1.09	1.1	0.85	0.70
Available phosphorus (%)	0.55	0.48	0.52	0.48	0.44	0.40
Sodium (%)	0.15	0.17	0.18	0.17	0.19	0.17
Methionine (%)	0.65	0.68	0.55	0.56	0.51	0.52
Lysine (%)	1.20	1.34	1.10	1.05	0.90	0.90
Methionine + cysteine (%)	0.90	0.92	0.81	0.79	0.75	0.74

a Concentrate *A*= in 1 kg of concentrate, 1300 kcal of metabolizable energy, 4.7 % lysine, 10.35 % methionine, 2.6 % threonine, 19.6 % calcium, 12.8 % available phosphorus, 2.9 % sodium and 4.2 % chlorine (0 to 24 days). Concentrate *B*= in 1 kg of concentrate, 1170 kcal of metabolizable energy, 5.2 % lysine, 9 % methionine, 2.8 % threonine, 21 % calcium, 12.2 % available phosphorus, 2.6 % sodium and 3.8 % chlorine (25 to 42 days).

b The following values are provided per kg of diet: vitamin A: 900 IU, vitamin D_3_: 2000 IU, vitamin E: 18 IU, vitamin K3: 2 mg, vitamin B_1_: 8.1 mg, vitamin B_2_: 6.6 mg, vitamin B_6_: 3 mg. vitamin B_12_: 15 µg, niacin: 30 mg, pantothenic acid: 10 mg, biotin: 1 mg, antioxidants: 100 mg, manganese: 100 mg, iron: 50 mg, zinc: 85 mg, copper: 10 mg, iodine: 5.1 mg, selenium: 2 mg.

### 2.2 Growth performance

To investigate the performance traits of growth, feed intake per experimental unit was measured in starter, grower, and finisher periods. The feed consumption of the experimental units was calculated based on the difference between the feed consumed at the end of the period and the allocated feed at the beginning of the period, on a per chick per day basis. Throughout the experimental period, the number of mortalities and their weights were recorded to account for the weight gain and feed consumption of the chicks that died during the experiment. The average body weight gain of the chicks in each replication was obtained by weighing the chicks in each cage at the beginning and end of the period. Prior to weighing the birds, the chicks were subjected to a four-hour deprivation of water and feed to ensure that their digestive system contents were consistent. The feed conversion ratio was calculated by dividing the feed consumed per chick in each period by the weight gain.

### 2.3 Carcass characteristics

To assess carcass characteristics, at the end of the experimental period (42 day), two chicks with weights close to the mean were selected, weighed, and then slaughtered. After slaughter and evisceration, the contents of the body were carefully removed. Subsequently, the weight of the carcass, total digestive system, liver, heart, abdominal fat, bursa of Fabricius, spleen, breast, and thigh were measured, and their relative weights were calculated as a percentage of live weight.

### 2.4 Nutrient digestibility

To assess nutrient digestibility, a 1 % acid insoluble ash (AIA) (a type of indigestible marker) was introduced into the bird's diet five days before the completion of both the starter period (at 6 day of age) and the grower period (at 20 day of age). The study on digestibility involved a three-days adaptation phase followed by a two-days collection of fecal samples. Following the removal of feathers and foreign substances from the samples, the excretas were placed into labeled containers and transported to the laboratory for further analysis. The feed and excreta samples were dried, ground, and stored at −20 °C until further analysis could be performed. Subsequently, the marker-labeled diet samples and bird excretas were analyzed for their composition (dry matter, organic matter, crude protein, and crude fat) using standard methods outlined by the Association of Official Analytical Chemists (2005). The concentration of acid insoluble ash in both the feed and excreta was determined using the procedure described by De Coca-Sinova et al. (2011). The equation used to calculate nutrient digestibility is as follows:

Digestibility(%)=[100−(100×AIAdiet/AIAfecal×Nutrientfecal/Nutrientdiet)]

### 2.5 Statistical analysis

The data were analyzed using the SAS software (version 9) in a completely randomized design (SAS, 2003). For the statistical model, the means were compared using a Tukey test at a significance level of 5 %. The control treatment was compared with other experimental treatments using independent comparisons at a significance level of 5 %.

## 3 Results and discussion

### 3.1 Growth performance

The impact of peppermint essential oil and artifier in low-energy diets on the growth performance of broiler chickens in the starter, grower, finisher, and overall periods is presented in Table 2. In the starter period, birds fed low-energy diets containing artifier and peppermint essential oil + artifier showed similar body weight gain and feed conversion ratio compared to birds fed the control diet. There was no significant difference in feed intake among the experimental treatments. In the grower period, birds fed low-energy diet containing peppermint essential oil + artifier exhibited similar body weight gain and feed conversion ratio compared to birds fed the control diet. Additionally, birds fed low-energy diet containing peppermint essential oil + artifier consumed less feed compared to birds fed low-energy diet containing peppermint essential oil alone (*P* < 0.05). In the finisher period, birds fed low-energy diet containing peppermint essential oil + artifier showed similar feed intake, body weight gain, and feed conversion ratio compared to birds fed the control diet. Furthermore, throughout the entire rearing period, birds fed low-energy diet containing peppermint essential oil + artifier exhibited similar body weight gain and feed conversion ratio compared to birds fed the control diet. There was no significant difference in feed intake among the experimental treatments.

**Table 2:** Effect of peppermint essential oil and artifier in low energy diets on growth performance of broiler chicken during different experimental periods.

Parameters	Control	Low energy diet	Low energy diet + Artifier	Low energy diet + PEO	Low energy diet + PEO + Artifier	SEM	*P*-value	Vs to control
Starter (0–10 days)								
Feed intake (g/bird/d)	29.2	29.6	29.5	29.3	29.4	0.67	0.99	0.74
Weight gain (g/bird/d)	22.0^a^	19.2^b^	21.4^a^	19.6^b^	22.3^a^	0.39	0.01	0.01
Food conversion ratio	1.32^b^	1.54^a^	1.38^b^	1.49^a^	1.32^b^	0.014	0.01	0.01
Grower (11–24 days)								
Feed intake (g/bird/d)	97.10^ab^	100.6^ab^	96.83^ab^	101.3^a^	95.70^b^	1.13	0.01	0.25
Weight gain (g/bird/d)	59.13^a^	54.42^bc^	55.86^bc^	53.95^c^	57.23^ab^	0.68	0.01	0.01
Food conversion ratio	1.64^c^	1.85^a^	1.73^b^	1.88^a^	1.67^c^	0.011	0.01	0.01
Finisher (25–42 days)							0.01	0.01
Feed intake (g/bird/d)	150.43^a^	142.91^b^	139.90^b^	142.73^b^	144.40^ab^	1.64	0.01	0.01
Weight gain (g/bird/d)	79.92^a^	66.75^c^	71.42^b^	67.94^bc^	78.12^a^	0.87	0.01	0.01
Food conversion ratio	1.88^c^	2.14^a^	1.95^b^	2.10^a^	1.85^c^	0.01	0.01	0.01
Total period (0–42days)								
Feed intake (g/bird/d)	103.80	100.94	99.04	101.60	100.80	1.1	0.09	0.02
Weight gain (g/bird/d)	59.20^a^	50.92^c^	54.21^b^	51.63^bc^	57.86^a^	0.65	0.01	0.01
Food conversion ratio	1.75^c^	1.98^a^	1.83^b^	1.97^a^	1.74^c^	0.008	0.01	0.01

^a,b,c^ Means within same row with different superscripts differ (*P* < 0.05). PEO: peppermint essential oil.

In a study conducted on broiler chicks, it was observed that consuming a diet containing 200 ppm carvacrol and thymol, which are derived from plants like oregano, peppermint, and thyme, led to a significant increase in feed intake and weight gain. This positive effect was attributed to the improved digestion and absorption of the feed within the chicks' digestive system, resulting in a better feed conversion ratio compared to the control treatment (Lee et al., 2003). Furthermore, the use of a 2 % combination of medicinal plants including peppermint, thyme, and savory demonstrated a significant improvement in the chicks' daily weight gain and feed conversion ratio when compared to the control treatment (Nobakht & Aghdam Shahriar, 2010). This improvement was attributed to the antibacterial and antifungal properties of the plant compounds used in the experimental treatments, which effectively reduced the population of harmful microbes in the chicks' digestive system. As a result, the health and safety of the chicks were enhanced, and the breakdown of proteins and amino acids by detrimental microbial populations was prevented, ultimately leading to an overall improvement in bird performance (Lee et al., 2003). Various species of mint plants are commonly utilized due to their abundant essential oils. Peppermint essential oils, for instance, have been shown to increase the length of the intestine and the surface area of contact between digested materials and the intestine. This expansion in contact surface provides greater opportunities for nutrient absorption (Alcicek et al., 2003). Menthol, which is found in peppermint, acts as a disinfectant for the digestive system, potentially reducing the presence of harmful microbes. Additionally, it stimulates the secretion of gastric juices and other digestive organs, leading to improved digestion and absorption of nutrients, ultimately resulting in enhanced growth performance (Motejaded et al., 2013). In a specific study, the feed conversion ratio of birds fed with 200 ppm peppermint essential oil and the antibiotic virginiamycin showed better results compared to those fed with control diets and 400 ppm peppermint essential oil (Khodambashi Emami et al., 2012).

In a research study involving broiler chicks fed with two different energy levels, the effects of incorporating emulsifiers, specifically lecithin and bile salts, in their diet were investigated (Guerreiro Neto et al., 2011). The findings indicated that the inclusion of emulsifiers resulted in increased feed intake, body weight, and feed conversion ratio. Additionally, the presence of emulsifiers contributed to improved production performance and carcass quality. These improvements can be attributed to the hydrophobic properties of compounds containing phospholipids, which have a strong affinity for combining with oils and fats in the small intestine. Consequently, emulsification is enhanced, leading to better nutrient absorption and overall enhancement of the chicks' performance (Guerreiro Neto et al., 2011). It is worth noting that young chicks experience a deficiency of bile acids in their digestive system during the first week of life. This deficiency could be due to inadequate production of bile acids in their bodies or their inability to effectively absorb them from the intestine. The addition of emulsifiers and bile salts can help address this deficiency and improve body weight gain and feed conversion ratio (Jamili et al., 2013). In another experiment, the use of lecithin did not have a significant impact on the final weight, daily weight gain at different stages of rearing, or mortality (Zampiga et al., 2016). However, variations in the results may arise from differences in control diets, types and levels of dietary fat, as well as the type and amount of emulsifiers used (Zhang et al., 2011). Kamran et al. (2020) demonstrated that increasing the level of polyglycerol polyricinoleate, an external emulsifier, in a diet containing soy oil as a fat source improved the feed conversion ratio and body weight during the starter phase and total peroid. Upadhaya et al. (2017) observed a strong positive correlation between the inclusion of external emulsifiers in broiler feed and body weight gain, as well as a strong negative correlation between the inclusion of external emulsifiers in broiler feed and feed conversion ratio. Emulsifier is known to improve performance by digestion of fats and support birds to overcome the inefficiency of lipase before 40 day of age in broilers (Tancharoenrat et al., 2013).

In the present experiment, the simultaneous use of peppermint essential oil and artifier in low-energy diets (150 kcal/kg lower than the recommended feeding guideline for Ross 308 strain) was able to achieve growth performance traits comparable to those observed in the control diet (with an energy level matching the requirements of Ross 308 strain).

### 3.2 Carcass characteristics

The effect of peppermint essential oil and artifier in low-energy diets on carcass performance and relative weights of internal organs in broiler chickens is presented in Table 3. The relative weights of the heart, spleen, bursa of Fabricius, carcass yield, thighs, and gastrointestinal tract were not affected by the experimental treatments. However, birds fed a low-energy diet containing artifier had lower relative liver weight compared to birds fed a low-energy diet containing peppermint essential oil (*P* < 0.05). Birds fed a low-energy diets containing artifier and peppermint essential oil + artifier had a lower percentage of abdominal fat compared to birds receiving a low-energy diet without additives and control birds (*P* < 0.05). Additionally, a similar percentage of breast was observed between control birds and birds fed low-energy diets containing peppermint essential oil + artifier and artifier.

**Table 3:** Effect of peppermint essential oil and artifier in low energy diets on carcass characteristics (% live body weight) of broiler chicken at 42 day.

Parameters	Control	Low energy diet	Low energy diet + Artifier	Low energy diet + PEO	Low energy diet + PEO + Artifier	SEM	*P*-value	Vs to control
Liver (%)	2.22^ab^	2.27^ab^	2.11^b^	2.36^a^	2.22^ab^	0.05	0.03	0.90
Heart (%)	0.53	0.50	0.52	0.55	0.53	0.02	0.67	0.54
Spleen (%)	0.110	0.115	0.108	0.106	0.108	0.02	0.94	0.86
Bursa of fabricius (%)	0.169	0.179	0.190	0.207	0.202	0.01	0.39	0.14
Carcass yield (%)	65.0	61.6	66.2	67.1	66.5	1.58	0.37	0.72
Abdominal fat (%)	1.83^a^	1.76^a^	1.38^b^	1.56^ab^	1.43^b^	0.07	0.01	0.01
Breast (%)	25.8^a^	21.9^b^	24.8^ab^	22.8^b^	24.9^ab^	0.75	0.02	0.01
Thigh (%)	18.0	18.6	19.5	18.9	19.6	0.59	0.60	0.19
Gastrointestinal tract (%)	7.10	7.14	7.14	7.06	7.11	0.25	0.99	0.82

^a,b,c^ Means within same row with different superscripts differ (*P* < 0.05). PEO: peppermint essential oil.

According to the results of the present study, it has been determined that the addition of phospholipids leads to a reduction in liver weight. Previous studies have shown that the liver plays a crucial role in fat metabolism in birds, and 95 % of fatty acid synthesis occurs in the liver. In diets with higher energy content, the excess energy is converted to fat by the liver, putting pressure on the liver and affecting its activity. Therefore, the reduction in liver weight and abdominal fat observed in this study might be attributed to a decrease in fat accumulation in the liver and abdominal region. Furthermore, lysophospholipids have the ability to modify circulation and facilitate the absorption of fat and protein, redirecting them towards muscle synthesis instead of fat deposition in the abdomen. This alteration affects the accumulation of fatty acids and amino acids in the meat produced by the birds (Chen et al., 2019). Consistent with the current study, a previous report indicated that adding 0.15 % lysophospholipids to the diet of broiler chickens had no impact on the relative weights of lymphoid organs but increased breast weight (Upadhaya et al., 2017). On the other hand, when 0.5 % emulsifier was added to diets with 150 kilocalories lower energy than the recommended commercial level, it led to an increase in spleen weight but had only a minor effect on carcass performance (Cho et al., 2012). Another experiment investigating the effect of adding lysophospholipids on carcass quality in broiler chickens found no significant differences among the experimental treatments in terms of carcass performance, breast weight, thighs, and wings. The discrepancies in the results regarding the addition of lysophospholipid supplements to the diet of broiler chickens may be attributed to variations in the source of the emulsifier and the composition of the control diet, which can have different impacts on carcass traits (Zampiga et al., 2016). Shahmoradi et al. (2022) showed the simultaneous utilization of 100 ppm l-carnitine and 1 g / kg lipidol (external emulsifier) in low-energy diet improved carcass quality, fat, protein and dry matter digestibility of broiler chickens

According to the findings of the current study conducted by Soltani and Nobakht (2015), the inclusion of peppermint, coriander, and satureja powders in the diet of broiler chickens did not result in any notable impact on the characteristics of the carcass. Similarly, Cabuk et al. (2006) found that the supplementation of herbal essential oil in the diet of broiler chickens did not lead to any discernible differences in various components of the carcass among different treatment treatments. However, it should be noted that the addition of plant essential oil did contribute to a reduction in the accumulation of abdominal fat, which is significant for maintaining consumer health and enhancing the quality of the carcass (Yoshioka et al., 2000). Furthermore, it has been reported that incorporating 30, 40, and 50 mL of wild mint extract into the diet of broiler chickens resulted in improved carcass characteristics. The disparities in the outcomes of studies may be attributed to variations in the plant species under investigation, variances in the content of active ingredients, and the quantity of extract utilized (Durrani et al., 2007). Lastly, the present study established that the addition of an emulsifier to the diet of broiler chickens can enhance carcass characteristics, whereas the inclusion of peppermint oil did not have a significant effect on carcass traits.

### 3.3 Nutrient digestibility

The impact of peppermint essential oil and artifier in low-energy diets on the digestibility of nutrients in broiler chickens during the starter and grower periods is presented in Table 4. Birds receiving peppermint essential oil and artifier in low-energy diets, either separately or in combination, had higher digestibility of dry matter and fat during the starter and grower periods compared to birds receiving low-energy diet without additives (*P* < 0.05). There was no significant difference in the digestibility of dry matter and fat between birds fed low-energy diet without additives and the control treatment. Also, there was no significant difference in the digestibility of protein and organic matter between the experimental treatments and the control treatment during the starter and grower periods.

**Table 4:** Effect of peppermint essential oil and artifier in low energy diets on nutrient digestibility (%) of broiler chicken at starter and grower periods.

Parameters	Control	Low energy diet	Low energy diet + Artifier	Low energy diet + PEO	Low energy diet + PEO + Artifier	SEM	*P*-value	Vs to control
Starter period								
Dry matter (%)	71.9^bc^	68.4^c^	75.4^ab^	76.3^a^	77.2^a^	0.90	0.01	0.03
Protein (%)	68.0	67.7	70.3	68.8	70.5	0.86	0.17	0.30
Fat (%)	73.0^bc^	71.4^c^	78.5^a^	76.8^ab^	80.2^a^	1.00	0.01	0.01
Organic matter (%)	71.2	70.3	72.2	71.8	72.5	0.70	0.18	0.78
Grower period								
Dry matter (%)	78.0^bc^	77.0^c^	81.8^ab^	80.3^ab^	82.5^a^	0.96	0.01	0.04
Protein (%)	65.7	64.1	65.4	63.7	64.9	0.92	0.49	0.20
Fat (%)	80.3^bc^	79.7^c^	85.2^a^	84.6^ab^	85.7^a^	0.99	0.01	0.01
Organic matter (%)	72.5	72.5	73.0	73.1	73.6	0.94	0.87	0.66

^a,b,c^ Means within same row with different superscripts differ (*P* < 0.05). PEO: peppermint essential oil.

In a study examining the impact of peppermint essential oil as a growth stimulant in broiler chickens, it has been reported that this essential oil significantly enhanced the digestibility of dry matter. Consequently, peppermint essential oil can be regarded as a growth stimulant owing to its enhanced digestibility. The advantageous effects of antioxidant compounds found in medicinal plants concerning the preservation of intestinal villi occur through cellular antioxidant activity. As a result, the antioxidant effects on intestinal villi enhance nutrient absorption. Furthermore, it has been reported that the active compounds present in peppermint essential oil serve as factors that improve digestibility, balance the intestinal microbial ecosystem, and stimulate the secretion of endogenous enzymes, thereby leading to improved growth performance in poultry)Khodambashi Emami et al., 2012). Researchers have attributed the positive effects of plant derivatives and herbal products on performance to factors such as their stimulatory impact on the digestive system and the process of digestion, the stimulation and augmentation of enzyme secretion, increased efficiency in the utilization of nutrients from feed, and improved liver function (Motejaded et al., 2013). In the current investigation, the addition of 150 ppm of peppermint essential oil to low-energy diets resulted in improved digestibility of dry matter and fat during the starter and grower periods. Shiri Ghzghapan et al. (2023) was reported that the simultaneous use of peppermint essential oil and artifier in low energy diets of broiler chickens compared to the control diet had the same performance and feed cost and improved blood biochemistry parameters and meat shelf life.

It has been well-established that the physiological ability of young chicks to digest fat is initially low but undergoes significant improvement from weeks 1.5 to 3. Consequently, numerous studies have focused on enhancing fat digestibility by providing bile acids and emulsifiers (Yoshioka et al., 2000). Furthermore, the inclusion of sodium stearoyl-2-lactylate supplement in low-energy diets has been found to enhance growth performance and counteract the adverse effects of reduced dietary energy levels. Young chicks fed diets containing higher levels of lysophospholipids exhibited greater digestibility of fat, dry matter, and metabolizable energy compared to diets without the supplement (Yoshioka et al., 2000). These findings align with the results of the present study, which demonstrated that the addition of 300 ppm of artifier to low-energy diets improved the digestibility of dry matter and fat during the starter and growth periods. The beneficial effects of artifier supplementation can be attributed to its emulsifying properties. Finally, the digestibility of dry matter and fat in low-energy diets containing a combination of peppermint essential oil and artifier was higher than in the control diet.

## 4 Conclusion

To summarize, the study found that the simultaneous use of 150 ppm peppermint essential oil and 300 ppm artifier in a low-energy diet (150 kilocalories per kilogram below the requirements of the Ross 308 strain) for broiler chickens had several positive effects. Firstly, it did not significantly affect weight gain and feed conversion ratio compared to the control diet (with energy levels matching the requirements of the Ross 308 strain). Moreover, the inclusion of these additives led to a decrease in abdominal fat and improved digestion of fat and dry matter in the chickens. The reduction in abdominal fat is desirable as it can enhance meat quality. The improved fat and dry matter digestion suggests that the additives help the chickens utilize nutrients more efficiently, resulting in better growth performance and feed efficiency. Overall, the study indicates that incorporating peppermint essential oil and artifier in broiler chicken diets can enhance growth, reduce abdominal fat, and improve nutrient utilization efficiency.

## Ethics statement

The authors confirm that the ethical policies of the journal, as noted on the journal's author guidelines page, have been adhered to and the appropriate ethical review committee approval has been received. The authors confirm that they have followed EU standards for the protection of animals used for scientific purposes.

## CRediT authorship contribution statement

**Shokoufe Ghazanfari:** Supervision. **Ayub Shiri Ghzghapan:** Investigation. **Shirin Honarbakhsh:** Writing – review & editing.

## Declaration of competing interest

The authors declare that they have no conflict of interest.
